# Correlation between Therapeutic Efficacy of CD34^+^ Cell Treatment and Directed *In Vivo* Angiogenesis in Patients with End-Stage Diffuse Coronary Artery Disease

**DOI:** 10.1155/2018/9591421

**Published:** 2018-04-15

**Authors:** Tien-Hung Huang, Cheuk-Kwan Sun, Yi-Ling Chen, Pei-Hsun Sung, Chi-Hsiang Chu, Mel S. Lee, Yuan-Ping Lin, Hon-Kan Yip, Fan-Yen Lee

**Affiliations:** ^1^Division of Cardiology, Department of Internal Medicine, Kaohsiung Chang Gung Memorial Hospital and Chang Gung University College of Medicine, Kaohsiung 83301, Taiwan; ^2^Institute for Translational Research in Biomedicine, Kaohsiung Chang Gung Memorial Hospital and Chang Gung University College of Medicine, Kaohsiung 83301, Taiwan; ^3^Department of Emergency Medicine, E-Da Hospital, I-Shou University School of Medicine for International Students, Kaohsiung 82445, Taiwan; ^4^Clinical Trial Center, Kaohsiung Chang Gung Memorial Hospital, Kaohsiung 83301, Taiwan; ^5^Department of Orthopedic Surgery, Kaohsiung Chang Gung Memorial Hospital and Chang Gung University College of Medicine, Kaohsiung 83301, Taiwan; ^6^Department of Health and Beauty, Shu-Zen Junior College of Medicine and Management, Kaohsiung 82144, Taiwan; ^7^Center for Shockwave Medicine and Tissue Engineering, Kaohsiung Chang Gung Memorial Hospital and Chang Gung University College of Medicine, Kaohsiung 83301, Taiwan; ^8^Department of Medical Research, China Medical University Hospital, China Medical University, Taichung 40402, Taiwan; ^9^Department of Nursing, Asia University, Taichung 41354, Taiwan; ^10^Division of Thoracic and Cardiovascular Surgery, Department of Surgery, Kaohsiung Chang Gung Memorial Hospital and Chang Gung University College of Medicine, Kaohsiung 83301, Taiwan; ^11^Tri-Service General Hospital, National Defense Medical Center, Taipei 11490, Taiwan

## Abstract

**Background:**

This study was aimed at testing the association between the therapeutic efficacy of CD34^+^ cell treatment in patients with end-stage diffuse coronary artery disease as reflected in angiographic grading and results of directed *in vivo* angiogenesis assay (DIVAA) on their isolated peripheral blood mononuclear cell- (PBMC-) derived endothelial progenitor cells (EPCs).

**Methods:**

Angiographic grades (0: <5%; 1: 5–35%; 2: 35–75%; 3: >75%) which presented the improvement of vessel density pre- and post-CD34^+^ treatment were given to 30 patients with end-stage diffuse coronary artery disease having received CD34^+^ cell treatment. The patients were categorized into low-score group (angiographic grade 0 or 1, *n* = 12) and high-score group (angiographic grade 2 or 3, *n* = 18). The percentages of circulating EPCs with KDR^+^/CD34^+^/CD45^−^, CD133^+^/CD34^+^/CD45^−^, and CD34^+^ were determined in each patient using flow cytometry. PBMC-derived EPCs from all patients were subjected to DIVAA through a 14-day implantation in nude mice. The DIVAA ratio (i.e., mean fluorescent units in angioreactors with EPCs/mean fluorescent units in angioreactors without EPCs) was obtained for each animal with implanted EPCs from each patient.

**Results and Conclusions:**

The number of EPCs showed no significant difference among the two groups. The DIVAA ratio in the high-score group was significantly higher than that in the low-score group (*p* = 0.0178). Logistic regression revealed a significant association between the DIVAA ratio and angiographic grading (OR 3.12, 95% CI: 1.14–8.55, *p* = 0.027). The area under the ROC curve (AUC) was 0.8519 (*p* = 0.0013). We proposed that DIVAA may be a reliable tool for assessing coronary vascularization after CD34^+^ cell treatment.

## 1. Introduction

The capability of bone marrow-derived CD34^+^ cells of vascular tissue repair and regeneration has been widely reported in a variety of animal disease models, including myocardial, peripheral, and cerebral ischemia [1, 2]. Besides, the clinical significance of CD34^+^ cell treatment has been highlighted in the promising results of a number of clinical trials enrolling patients with ischemic diseases unsuitable for surgery or refractory to medications [3–8]. Although the increasing number of successful bench-to-bedside (i.e., phase 1) studies has provided enough evidence to support the clinical safety of autologous stem cell treatment, the selection of optimal time points, cell dosage, and route of administration remains unclear [9, 10].

In our previous clinical trial [6], patients with severe diffuse coronary artery disease (CAD) receiving intracoronary transfusion of circulating-derived CD34^+^ cells directly without enrichment for endothelial progenitor cells (EPCs) were divided into angiographic low- and high-score groups after a 9-month follow-up. The results of cardiac MRI and 3D echocardiography demonstrated a significant improvement of the left ventricular ejection fraction (LVEF) in the high-score subjects compared to that in their low-score counterparts. The findings, therefore, indicated that angiographic grading could reliably reflect the long-term outcome of patients after CD34^+^ cell treatment. On the other hand, since up to 31.6% of patients (12 of 38) responded unsatisfactorily to cell treatment in that study, prediction of treatment outcome with angiographic grading may be of clinical importance for selection of patients for advanced stem cell treatment, such as enrichment for EPC purity and quantity in Good Tissue Practice (GTP) facilities.

The present study attempted to investigate the association of angiographic grading with the results of directed *in vivo* angiogenesis assay (DIVAA) in patients with end-stage diffuse coronary artery disease having received CD34^+^ cell treatment.

## 2. Subjects and Methods

### 2.1. Study Populations and Study Protocol

Between February 2015 and March 2016, all patients of age between 20 and 80 having received CD34^+^ cell treatment (phase I clinical trial) for obstructive coronary disease (including stable and unstable angina and ≥3 months of myocardial infarction ≤ Killip-3) with coronary angiographic findings of severe diffuse CAD, noncandidates for percutaneous coronary intervention or coronary artery bypass surgery (CABG) (i.e., when vessel involvement was too diffuse and the diameter was too small for intervention), those who had Canadian Cardiovascular Society class II–IV angina, and those whose thallium (Tl-201) scan showed reversible myocardial ischemia as well as those who had previously received CABG with venous or arterial graft failure that could not be revascularized by the catheter-based approach for either the native vessel or the graft were prospectively enrolled. Patients who were carriers of hepatitis B or C; those with history of surgery, trauma, or myocardial infarction within the preceding 3 months; those with liver cirrhosis, hematology disorders, renal insufficiency (defined as creatinine clearance [Ccr] < 20 mL/min), malignancy, febrile disorders, acute or chronic inflammatory disease at study entry, severe mitral or aortic regurgitation, congestive heart failure (NYHA Fc 4), expected life expectancy less than 2.0 years, and age younger than 20 years or 80 years or older; or pregnant women were excluded from the present study. A total of 38 patients were enrolled in our previous phase I clinical trial, and results have been published [6]. However, three patients expired due to cardiovascular death and five patients expired due to noncardiovascular death in the period of long-term follow-up. As a result, only 30 of the 38 patients participated in the present study.

**Figure 1:**
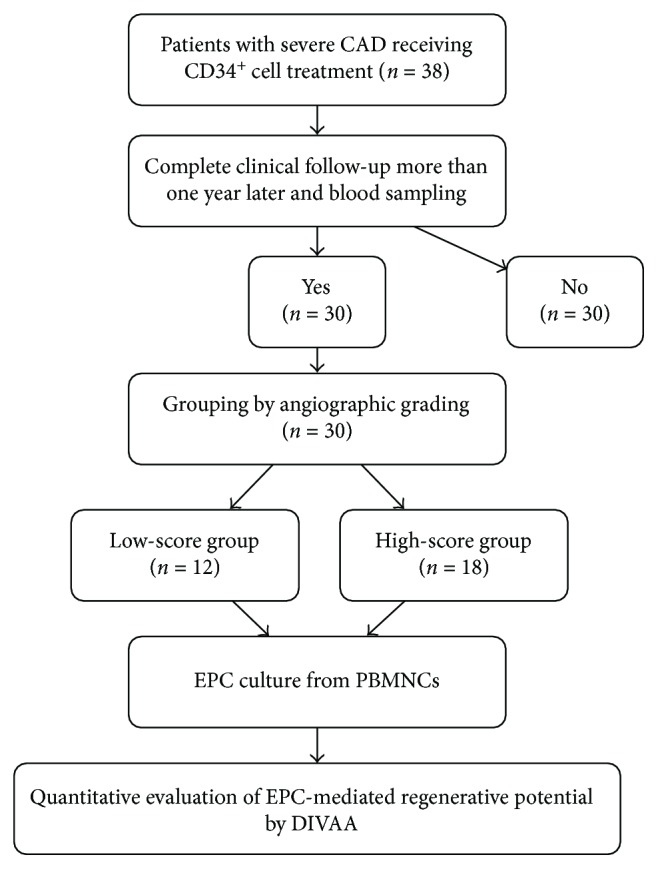
Protocol of follow-up, grouping, and analysis in the present study. Patients with severe coronary artery disease (CAD) having received CD34^+^ cell treatment with complete follow-up, divided into three groups based on different angiographic grades. Application of directed *in vivo* angiogenesis assay (DIVAA) further delineating the correlation between angiographic grading and endothelial progenitor cell- (EPC-) mediated regenerative potential.

All eligible patients having received CD34^+^ cell treatment for over one year underwent peripheral blood sampling in the outpatient clinic of a tertiary referral center (Kaohsiung Chang Gung Memorial Hospital) for the culturing of endothelial progenitor cells (EPCs). Quantitative assessment of angiogenic responses in the cultured EPCs was performed by directed *in vivo* angiogenesis assay (DIVAA) using nude mice. Patients with incomplete clinical follow-up were excluded. An angiographic study of the coronary arteries was performed nine months after cell therapy. The degree of angiogenesis on angiography was compared to that of DIVAA for each patient. An overview of the study protocol is shown in Figure 1. This study was approved by the Institutional Review Committee on Human Research at Chang Gung Memorial Hospital (IRB No.: 103–7569A3) and conducted at Kaohsiung Chang Gung Memorial Hospital.

### 2.2. Definition of Angiographic Grading

Improved angiogenesis/neovascularization for patients pre- and post-autologous CD34^+^ cell treatment was assessed angiographically using the scoring system of vessel density previously described [3]. Briefly, four cardiologists blinded to the treatment had reached a consensus for grading with scores being defined as follows: grade 0: less than 5.0%; grade 1: 5.0% to 35.0%; grade 2: greater than 35.0% to less than or equal to 75.0%; and grade 3: greater than 75.0%. Because of the limited number of patients for each angiographic grade (i.e., 0, 1, 2, and 3), the patients were divided into low- and high-score groups. High-score was defined as grade 2 and grade 3, while low-score was defined as grade 0 and grade 1.

### 2.3. Flow Cytometry Analysis

For identification of EPCs derived from peripheral blood, flow cytometry was applied for the assessment of EPC surface markers, including sets of KDR^+^/CD34^+^/CD45^−^, CD133^+^/CD34^+^/CD45^−^, and CD34^+^, as described in our recent reports [6, 8, 11]. Briefly, mononuclear cells were isolated by density-gradient centrifugation of Ficoll-Paque Plus™ (GE Healthcare Biosciences, Uppsala, Sweden) and further incubated with fluorescent dye-conjugated mononuclear antibodies. After incubation, the mononuclear cells were fixed in 1% of paraformaldehyde for flow cytometry analysis by using a fluorescence-activated cell sorter (FACSCalibur system; Beckman Coulter, CA, USA). The assays for circulating EPCs in each sample were performed in duplicate, and the mean levels were reported.

### 2.4. Endothelial Progenitor Cell Culture

Ficoll gradient was used to isolate peripheral blood mononuclear cells (PBMCs) from blood collection in heparin tubes for the culture of endothelial progenitor cells (EPCs), and the protocol of human EPC culture has been described in our previous studies [6, 8]. In brief, whole blood was diluted (1 : 1) with PBS containing 2% fetal bovine serum (FBS; Gibco BRL, CA, USA), and 7 mL of diluted blood was then carefully overlaid onto 5 mL of Ficoll-Paque Plus (GE Healthcare Biosciences, Uppsala, Sweden). The PBMC layer was isolated by centrifugation without a brake and washed with PBS. Isolated PBMCs were cultured with the endothelial growth medium-2 (EGM-2; Clonetics®, CA, USA) on fibronectin-coated dish at 37°C with 5% CO_2_.

### 2.5. Directed *In Vivo* Angiogenesis Assay (DIVAA)

**Figure 2:**
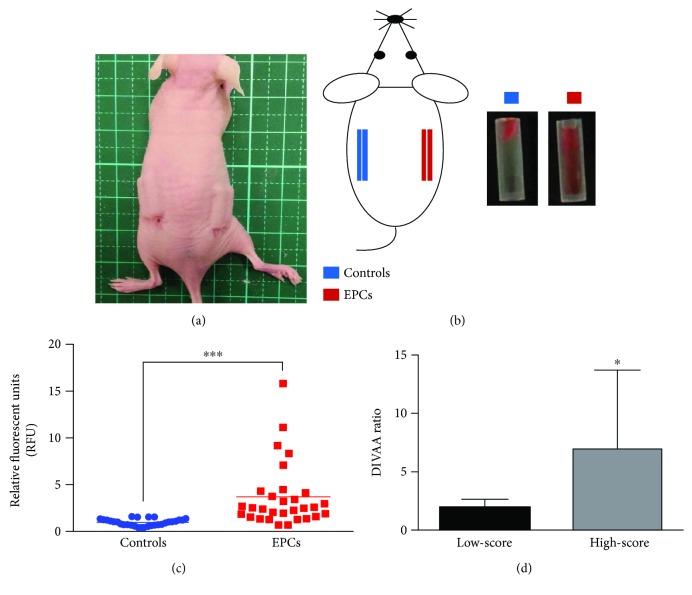
Evaluation of endothelial progenitor cell- (EPC-) mediated angiogenesis/neovascularization by directed *in vivo* angiogenesis assay (DIVAA) in nude mice. (a) Angioreactors implanted subcutaneously on each flank of a nude mouse. (b) Two angioreactors containing growth factors with (i.e., right side, EPCs) and two without (i.e., left side, controls) cultured EPCs (4 × 10^5^ EPCs/angioreactor) from one patient subcutaneously implanted under the surgical incisions. After a 14-day implantation, retrieved angioreactors displayed on the right panel. (c) Comparison of relative fluorescent units (RFUs) between controls and EPCs with the paired *t*-test. (d) Comparison of the DIVAA ratio between the low-score group (grades 0-1) and the high-score group (grades 2-3), using the independent *t*-test; each error bar represents the mean with SD, ^∗∗∗^*p* < 0.0001 and ^∗^*p* < 0.05.

Angiogenic responses of EPCs of each patient were quantitatively assessed by directed *in vivo* angiogenesis assay (DIVAA) in a nude mouse according to the protocol of the manufacturer (Trevigen, MD, USA). Briefly, female nude mice, 6 to 8 weeks of age, were anesthetized with 2% isoflurane for the implantation of the angioreactor. Two incisions were made on the dorsal-lateral skin of a nude mouse, one on each side, approximately 1 cm in length. Two angioreactors containing growth factors with (i.e., right side) and two without (i.e., left side) cultured EPCs (4 × 10^5^ EPCs per angioreactor) from one patient were subcutaneously implanted under the surgical incisions in one nude mouse which were closed with interrupted 4-0 nylon sutures (Figures 2(a) and 2(b)). After 14 days, the 30 nude mice carrying EPCs from 30 patients were anesthetized with 2% isoflurane before careful removal of the implanted angioreactors. Cells in the angioreactor were collected for incubation with fluorescein isothiocyanate- (FITC-) conjugated lectin at 4°C overnight. Vascularization in the angioreactor was assessed with a spectrophotometer (Thermo Scientific™ Fluoroskan Ascent™ FL Microplate Fluorometer, Vantaa, Finland) at 485 nm based on the degree of specific binding between lectin and endothelial cells as reflected in the intensity of FITC-lectin fluorescence signals (i.e., relative fluorescent units, RFU). After a 14-day implantation, the level of angiogenesis and neovascularization as reflected in the mean fluorescence intensity in the two angioreactors on the right side (i.e., EPCs) to that in the other two on the left side (i.e., controls) was compared using the DIVAA ratio (i.e., mean fluorescent units in angioreactors with EPCs/mean fluorescent units in angioreactors without EPCs) for each animal. All animal experimental procedures were approved by the Institute of Animal Care and Use Committee at Kaohsiung Chang Gung Memorial Hospital (Affidavit of Approval of Animal Use Protocol No. 2014121814) and performed in accordance with the National Research Council of the National Academies Guide for the Care and Use of Laboratory Animals (Eighth Edition, 2011).

### 2.6. Statistical Analysis

All values are expressed as the mean ± SD, number, or percentage where appropriate. Differences in continuous variables between two groups were analyzed by the independent *t*-test. The means of more than three independent groups were analyzed by ANOVA. Continuous variables were compared with a paired *t*-test for matched-paired samples. The Pearson correlation test was used to assess the relation between two quantitative variables. A binary logistic regression model was further used to evaluate the major factors related to angiogenesis grading. Statistical analysis was performed using SPSS statistical software for Windows version 13 (SPSS for Windows, version 13; SPSS, Chicago, IL). A value of *p* less than 0.05 was considered statistically significant.

## 3. Results

### 3.1. Characteristics of Study Patients

Of the 38 patients with severe diffuse CAD having undergone CD34^+^ cell treatment, eight of the patients expired in the long-term follow-up. As a result, only 30 of the 38 patients participated in the present study (Figure 1). According to follow-up angiography grading, CAD patients were categorized into the low-score group (angiographic grade 0 or 1, *n* = 12) and high-score group (angiographic grade 2 or 3, *n* = 18). The characteristics of the 30 patients are summarized in Table 1. The age, gender, body height, body weight, body mass index, CAD risk factors, and incidences of previous stroke, old myocardial infarction, and coronary artery bypass surgery did not differ between low-score and high-score group patients. Additionally, findings of vessel diseases and use of medication did not show a significant difference among these groups. The laboratory findings are also summarized. The serum creatinine level, creatinine clearance rate, and incidence of moderate and severe chronic kidney disease (CKD, stage III, and stage IV) did not differ among the three groups. For the percentages of circulating cells with KDR^+^/CD34^+^/CD45^−^, CD133^+^/CD34^+^/CD45^−^, and CD34^+^, the three stem cell marker sets showed no difference among the three groups.

### 3.2. *In Vivo* Angiogenic Potential of EPCs Determined by DIVAA

Comparison of the fluorescence intensities of the 30 mice on both sides revealed significantly higher relative fluorescent units (RFU) on the right side (i.e., EPCs) compared to that on the left side (i.e., controls) (*p* < 0.0001, Figure 2(c)). To clarify whether capacities of angiogenesis and neovascularization of EPCs isolated from patients varied with their angiographic grades, EPCs were divided into low-score group (angiographic grade = 0 or 1, *n* = 12) and high-score group (angiographic grade = 2 or 3, *n* = 18) for comparison. Comparison of the DIVAA between the two groups demonstrated that the DIVAA ratio of the high-score group was significantly higher than that in the low-score group (*p* = 0.0178) (Figure 2(d)).

### 3.3. Association of the DIVAA Ratio with Angiographic Grading

To study the association between the DIVAA ratio and angiographic grading, both univariate and multivariate logistic regression models were used to find the relationships between the important variables and angiographic findings. The univariate logistic regression analysis was applied to select the significant variable (*p* value < 0.05) (Table 2). When the DIVAA ratio was used as the predictive tool for the angiographic findings after receiving autologous CD34^+^ cell treatment, patients with a higher DIVAA ratio had a higher angiogenesis/neovasculature score (OR 3.12, 95% CI: 1.14–8.55, *p* = 0.027).

### 3.4. Predictive Power of the DIVAA Ratio

**Figure 3:**
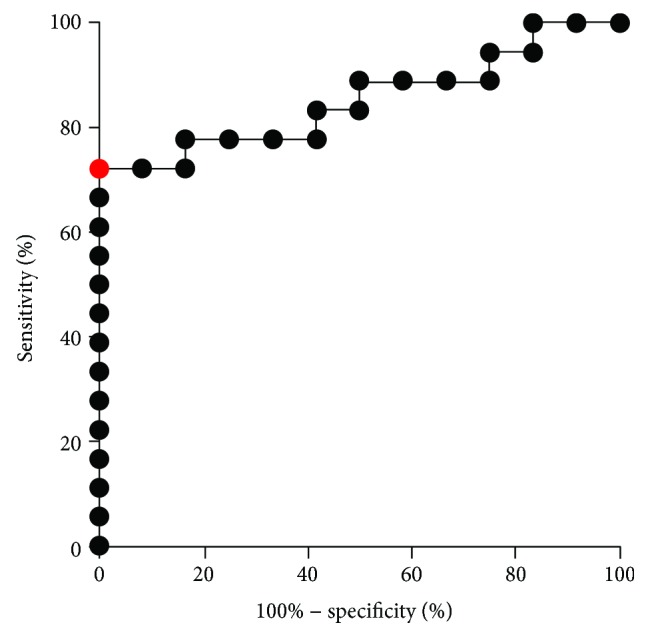
Association between angiographic findings and the DIVAA ratio. Assessment of the receiver operator characteristic (ROC) curve for the predictive power of the directed *in vivo* angiogenesis assay (DIVAA) ratio in patients having received autologous CD34^+^ cell treatment. The area under the ROC curve (AUC) was 0.8519 (*p* = 0.0013). The optimal cut-off point is labeled by the red solid circle.

To clarify the power of DIVAA in predicting angiographic outcomes of patients having received autologous CD34^+^ cell treatment, receiver operator characteristic (ROC) curve analysis was performed (Figure 3). When comparison was made between low and high angiographic scores, the area under the ROC curve (AUC) was 0.8519 (95% CI: 0.7132–0.9905, *p* = 0.0013). An optimal cut-off point at 3.07 was determined using the ROC curve. When patients were divided according to the cut-off point, sensitivity and specificity of the high score were 72.22% and 100%, respectively. Furthermore, if we want to correctly identify the high score of patients (more than 85%), an appropriate value of the cut-off point was considered to be 1.965 with sensitivity 88.9% and specificity 50.0%, respectively.

## 4. Discussion

This study, which attempted to investigate the correlation between angiogenesis/neovascularization of EPCs from patients with severe diffuse coronary artery disease having received autologous CD34^+^ cell treatment and their angiographic grading, demonstrated that higher DIVAA ratios were associated with higher angiographic grading. The present investigation represents the first study to address the possibility of using a DIVAA murine model to correlate with clinical outcomes.

CD34^+^ is the most commonly used marker of endothelial progenitor cells (EPCs) in clinical hematology and stem cell trials [4, 6, 12]. Although the capacity of transdifferentiation in hematopoietic stem cells and EPCs to cardiac myocytes has been widely debated [13, 14], neovascularization is still the most important outcome after receiving CD34^+^ cell treatment through direct incorporation of transfused cells into newly formed vessels and release of angiogenic cytokines in a paracrine manner [1, 15]. Since we found that many baseline characteristics and routine laboratory findings cannot clearly explain the changes in angiographic grading in advance, we focused on the investigation of the regenerative potential of isolated CD34^+^ cells that may reflect the neovascularization of coronary vasculature in the present study.

Tube formation assay is a regular angiogenesis assay widely applied to determine the angiogenic potential of EPCs *in vitro*. However, our previous study has demonstrated that there is no significant difference in EPC-mediated angiogenesis between low-score and high-score groups before G-CSF (granulocyte colony-stimulating factor) stimulation by tube formation assay [6]. There are three merits of using DIVAA to replace the conventional tube formation assay to determine EPC-mediated angiogenesis/neovascularization in this study. First, DIVAA more closely mimics the actual physiological environment of the human body. Second, DIVAA allows long-term observation for 9 to 15 days to enable a more accurate assessment of vascularization. In contrast, the capillary-like structure in tube formation assay, which is usually observed after a 6- to 12-hour incubation, invariably disappears on prolonged incubation so that long-term observation of vascularization is impossible. Third, the outcome of conventional tube formation assay that depends on cell density and cellular migration capacity [16, 17] cannot reliably assess EPC-mediated neovascularization, which is mainly attributable to the differentiation from EPCs to mature endothelial cells [18]. Therefore, not only does DIVAA provide a physiological environment for EPC differentiation to mature endothelial cells, but it also allows long-term observation of angiogenesis/neovascularization.

The therapeutic benefits of administering CD34^+^ cells in patients with ischemic heart disease are believed to be attributable to the angiogenic capacity of EPCs. The discrepancy in treatment outcome of using CD34^+^ cells in previous studies may be attributable to the fact that EPCs comprise only a minor proportion of CD34^+^ cells that also include several subpopulations of hematopoietic stem cells [19–21]. Indeed, it has been demonstrated that EPCs account for merely 1% of all circulating mononuclear cells [22]. Therefore, direct transfusion of isolated CD34^+^ cells might not guarantee sufficient purity of EPCs to achieve an optimal therapeutic outcome. On the other hand, a sufficient quantity of EPCs may not attain the expected treatment goal since the angiogenic capacity of EPCs, including proliferation, differentiation, and mobilization in bone marrow, may be tarnished by the presence of chronic diseases like diabetes, hypertension, and renal dysfunction that often cause systemic oxidative stress [23, 24]. Hence, the quality of stem cells for clinical application should be taken into consideration [21]. For this purpose, a long-term evaluation of *in vivo* angiogenic potential by DIVAA might help in differentiating the capacity of EPC-mediated angiogenesis/neovascularization in patients with different underlying diseases.

As stem cell therapy has been gaining popularity as a standard treatment option, the concept of improving such a “stem cell drug” product, such as safety, reproduction, and efficiency, has been developed [25]. The results of the present study demonstrated a significant correlation between the DIVAA ratio and clinical angiographic grading, suggesting the possibility of using this ratio as guidance for choosing the subsequent treatment strategy. For example, patients with a high DIVAA ratio may be suggested to receive autologous CD34^+^ cells directly with minimum manipulation, whereas patients with a low DIVAA ratio may receive EPCs enriched in good tissue practice (GTP) facilities before intracoronary transfusion. Although most of the stem cell-based clinical trials focused on the investigation of the optimal routes, dosage, and timing for stem cell administration, the results of the current study suggest that determination of the quality and quantity of stem cells is necessary to achieve an optimal therapeutic outcome for an individual based on the concept of precision medicine that emphasizes on tailoring medical care to cater for individual needs [26].

## 5. Limitations

This study has limitations. First, the sample size was relatively small in the present study. As a result, patients from our phase II and III clinical trials will be enrolled in the future to reinforce our findings. Second, since EPCs were not isolated from the study subjects before CD34^+^ cell treatment, the value of DIVAA in predicting the subsequent angiographic grading remains unclear. Third, it is hard to distinguish whether EPC-directed angiogenesis contributed by paracrine effect of proangiogenic factors or direct incorporation into new vessels in our DIVAA model.

## 6. Conclusions

The results of the present study demonstrated a significant correlation between EPC angiogenic capacity from patients with end-stage diffuse coronary artery disease in an *in vivo* murine model and their angiographic grading, suggesting the possible use of this model in the prediction of clinical outcome after CD34^+^ cell treatment.

## Figures and Tables

**Table 1:** Clinical and laboratory findings of 30 study patients.

Variables	Low-score (*n* = 12)	High-score (*n* = 18)	*p* value
Age (years)	65.3 ± 9.8	66.0 ± 6.3	0.799
Male gender	58.3% (7)	72.2% (13)	0.429
Body height (cm)	158.8 ± 8.1	161.9 ± 6.2	0.243
Body weight (kg)	67.4 ± 14.4	70.0 ± 10.2	0.200
Body mass index	26.1 ± 4.7	26.7 ± 3.7	0.692
Hypertension	91.7% (11)	83.3% (15)	0.632
Diabetes mellitus	75.0% (9)	83.3% (15)	0.660
History or current smoking	33.3% (4)	50.0% (9)	0.367
Total cholesterol (mg/dL)	182.1 ± 36.3	164.4 ± 42.1	0.246
Low density lipoprotein	102.9 ± 25.5	106.0 ± 40.1	0.815
High density lipoprotein	47.6 ± 13.1	42.8 ± 8.5	0.238
Previous stroke	16.7% (2)	22.2% (4)	1.000
Old myocardial infarction	16.7% (2)	16.7% (3)	1.000
History of CABG	41.7% (5)	33.3% (6)	0.643
Diffuse multivessel disease	100% (12)	100% (18)	1.000
Diffuse triple vessel disease	83.3% (10)	83.3% (15)	1.000
Previous PCI	58.3% (7)	44.4% (8)	0.456
Aspirin/clopidogrel therapy	100% (12)	100% (18)	1.000
*β*-Blocker therapy	91.7% (11)	94.4% (17)	1.000
Stain therapy	58.3% (7)	77.8% (14)	0.255
ARB/ACEI therapy	75.0% (9)	77.8% (14)	1.000
Creatinine level (mg/dL)	1.08 ± 0.31	1.26 ± 0.42	0.391
Creatinine clearance rate (mL/min)	64.8 ± 17.7	59.9 ± 23.1	0.539
Stage III–IV CKD	58.3% (7)	55.6% (10)	0.880
Flow cytometry (before G-CSF treatment)			
KDR^+^/CD34^+^/CD45^−^ (%)	0.22 ± 0.19	0.24 ± 0.14	0.430
CD133^+^/CD34^+^/CD45^−^ (%)	0.12 ± 0.11	0.08 ± 0.05	0.458
CD34^+^ (%)	1.19 ± 0.91	1.05 ± 1.02	0.711
Flow cytometry (after G-CSF treatment)			
KDR^+^/CD34^+^/CD45^−^ (%)	0.52 ± 0.67	0.34 ± 0.55	0.325
CD133^+^/CD34^+^/CD45^−^ (%)	1.10 ± 0.87	0.75 ± 0.52	0.347
CD34^+^ (%)	2.42 ± 2.08	1.88 ± 1.65	0.879

Data are expressed as mean ± SD or % (*n*). CABG = coronary artery bypass grafting; PCI = percutaneous coronary intervention; ARB = angiotensin II type I receptor blocker, ACEI = angiotensin-converting enzyme inhibitor; CKD = chronic kidney disease.

**Table 2:** Univariate logistic regression models for the association between significant variables and improved angiographic findings.

Univariate logistic regression model			
Variables	OR	95% CI	*p* value
Age	1.01	(0.92–1.12)	0.791
Gender	1.86	(0.40–8.69)	0.432
Body height	1.07	(0.96–1.20)	0.237
Body weight	1.02	(0.96–1.09)	0.557
Body mass index	1.04	(0.86–1.25)	0.706
Hypertension	0.46	(0.04–4.98)	0.518
Diabetes mellitus	1.67	(0.28–10.09)	0.578
History or current smoking	2.00	(0.44–9.10)	0.370
Total cholesterol	0.99	(0.97–1.01)	0.242
Low-density lipoprotein	1.00	(0.98–1.03)	0.808
High-density lipoprotein	0.96	(0.89–1.03)	0.236
Previous stroke	1.43	(0.22–9.38)	0.710
Old myocardial infarction	1.00	(0.14–7.10)	1.000
History of CABG	0.70	(0.16–3.17)	0.643
Diffuse multivessel disease	—	—	—
Diffuse triple vessel disease	1.00	(0.14–7.10)	1.000
Previous PCI	0.57	(0.13–2.50)	0.458
Aspirin/clopidogrel therapy	—	—	—
*β*-Blocker therapy	0.77	(0.09–27.36)	0.767
Stain therapy	2.50	(0.51–12.35)	0.261
ARB/ACEI therapy	1.17	(0.21–6.48)	0.860
Creatinine level	3.79	(0.43–33.72)	0.232
Creatinine clearance rate	0.99	(0.95–1.02)	0.525
Stage III–IV CKD	0.89	(0.20–3.91)	0.880
Before G-CSF treatment			
KDR^+^/CD34^+^/CD45^−^	2.26	(0.02–319.53)	0.747
CD133^+^/CD34^+^/CD45^−^	0.001	(0.00–118.39)	0.240
CD34^+^	0.85	(0.39–1.89)	0.696
After G-CSF treatment			
KDR^+^/CD34^+^/CD45^−^	0.58	(0.16–2.13)	0.409
CD133^+^/CD34^+^/CD45^−^	0.46	(0.14–1.49)	0.197
CD34^+^	0.85	(0.56–1.28)	0.430
DIVAA ratio	3.12	(1.14–8.55)	0.027^∗^

^∗^Defined significant at *α* = 0.05.
